# Associations between dietary intake of live microorganisms, fermented foods, and the fermented food microbial richness index and cardiometabolic health in Swiss adults: a cross-sectional analysis

**DOI:** 10.1007/s00394-026-03895-y

**Published:** 2026-02-12

**Authors:** Eugenia Pertziger, Elaine Hillesheim, Olivier Bonny, Carsten A. Wagner, Guy Vergères, Murielle Bochud, Kathryn J. Burton-Pimentel

**Affiliations:** 1 Agroscope, Food Microbial Systems Research Division, Bern, Switzerland; 2 Department of Epidemiology and Health Systems, Center for Primary Care and Public Health (Unisanté), University of Lausanne, Lausanne, Switzerland; 3 Service of Nephrology, Fribourg State Hospital and University of Fribourg, Fribourg, Switzerland; Service of Nephrology, Lausanne University Hospital, Lausanne, Switzerland; 4 Institute of Physiology and Zurich Kidney Center, University of Zurich, Zurich, Switzerland

**Keywords:** Dietary live microbes, Fermented foods, Food microbiota, Food microbial richness index, Switzerland, Dietary intake, 24-h dietary recalls

## Abstract

**Purpose:**

Dietary microorganisms are hypothesised to contribute to human health by modulating gut microbiota composition and function. However, evidence of their impact on health at the level of the overall diet is still emerging. In this study, we examined associations of dietary intake of live microorganisms, fermented foods, and microbial richness with cardiometabolic health.

**Methods:**

We analysed baseline data of 440 adults (≥ 18 years) in the Swiss Kidney Stone Cohort (SKSC). Foods were categorised by live microorganism levels (low, < 10^4^ CFU/g; medium [Med], 10^4^–10⁷ CFU/g; or high [Hi], > 10⁷ CFU/g) and fermented food descriptors, including core microbiota. A microbial richness index was calculated based on the number of unique microbial species in fermented foods. Adjusted linear regression models assessed associations of MedHi food (> 10^4^ CFU/g) intake, fermented food intake, and the fermented food microbial richness index with cardiometabolic parameters.

**Results:**

Higher intake of microbe-rich MedHi foods was associated with lower diastolic blood pressure (β = − 1.99 × 10^–2^; CI − 3.71 × 10^–2^, − 2.82 × 10^–3^; *p* = 0.02), with fruit and vegetable consumption influencing this relationship. Total fermented food intake was not associated with any of the cardiometabolic parameters, but a higher value for the microbial richness index was associated with lower systolic blood pressure (β = − 2.07 × 10^–3^; CI − 3.85 × 10^–3^, − 2.95 × 10^–4^; *p* = 0.02) and glycated haemoglobin (β = − 1.22 × 10^–5^; CI − 2.14 × 10^–5^, − 3.11 × 10^–6^; *p* = 0.01), independent of any food subgroups.

**Conclusion:**

Greater diversity of microbes from fermented foods was associated with lower systolic blood pressure and glycated haemoglobin in this study, suggesting potential cardiometabolic benefits.

**Supplementary Information:**

The online version contains supplementary material available at 10.1007/s00394-026-03895-y.

## Introduction

Cardiometabolic diseases (CMDs)—a group of interconnected disorders including obesity, diabetes, heart disease, and stroke—have become a leading cause of morbidity and mortality globally [1]. These conditions are linked to a complex interplay of non-modifiable genetic predisposition and modifiable environmental and lifestyle factors such as physical inactivity, smoking, and unhealthy diets [2]. The gut microbiota has emerged as a potential mediator of the relationship between diet and the development and progression of CMDs, influencing host metabolism, inflammation, and vascular function [3–8]. Since the gut microbiota can be modified through dietary interventions [9, 10], understanding the relationship between diet and the gut microbiota is important for supporting the prevention and treatment of CMDs.

Among the most prominent diet-based strategies to modulate the gut microbiota, the direct delivery of non-pathogenic microorganisms via fermented dairy foods and probiotics has been widely investigated in CMDs with some promising results [11]. However, diet comprises multiple sources of microorganisms that may affect CMDs. Interestingly, the importance of previously overlooked sources of dietary microorganisms that are captured by the live dietary microbes classification, such as those derived from fruits and vegetables, has been corroborated by recent evidence showing that these microbes directly impact the gut microbiome [12].

To capture different diet-derived microbes, the total intake of live dietary microorganisms was recently estimated in the US population by categorising all foods recorded in the National Health and Nutrition Examination Survey (NHANES) into three levels of live microbes: low, medium, and high [13]. Within this dataset, higher intake of live microorganisms has been linked to several positive health outcomes, including improved cardiometabolic health parameters [14], lower prevalence of metabolic syndrome [15] and insulin resistance [16], lower risk of all-cause and cardiovascular disease mortality [17].

Similarly, we recently analysed the diet of Swiss adults, categorising foods by the level of live microorganisms, as previously described [18]. We further classified foods into fermented and non-fermented categories and identified their core microbiota. This comprehensive approach assessed both the quantity of these foods consumed and the diversity of live microorganisms in the diet. It also established a foundation for examining the relationship between these dietary factors and cardiometabolic health in a European context. To achieve this, we applied our dietary microbes classification to the Swiss Kidney Stone Cohort (SKSC) and conducted regression analyses to evaluate the associations between dietary live microbes, fermented foods, and the fermented food microbial richness index and physiological and anthropometric parameters related to cardiometabolic health.

## Methods

### Study population

We used cross-sectional baseline data from the SKSC, a multicentre cohort of incident and recurrent kidney stone formers, and a control group of computed tomography (CT) proven stone non-formers [19, 20], which makes it a hybrid cohort design with embedded controls. The study involved six centres in the German- and French-speaking parts of Switzerland: Zurich, Basel, Aarau, Bern, Lausanne, and Geneva. Stone formers were recruited at nephrology outpatient clinics. Inclusion criteria for the cases were written informed consent, age ≥ 18 years, and either recurrent kidney stone episodes (> 1) or a single kidney stone episode combined with at least one of the additional risk factors for stone recurrence, as detailed in the study protocol [19, 21]. The control group of stone non-formers was recruited from the general adult population through advertisements in Geneva, Lausanne, Aarau, and Zurich. Inclusion criteria for the controls were written informed consent, age ≥ 18 years, and confirmation of stone-free status on a low-dose CT scan. Participants were enrolled between April 2014 and March 2020, with the last 3-year follow-up taking place in spring 2023.

At the baseline visit, participants underwent a comprehensive assessment that included collection of demographic and anthropometric data, medical and kidney stone history, physical examination, two consecutive 24-h dietary recalls, two parallel consecutive 24-h urine samples, and blood samples. The 24-h dietary recalls were conducted by trained dieticians, using the validated software GloboDiet® (version CH-2016.4.10, International Agency for Research on Cancer) [22–24]. The dietary data were then linked to the Swiss food composition database using FoodCASE® (version 8.3.0, Premotec GmbH) to estimate macro- and micronutrient intakes. For the present analysis, we used baseline data from participants (cases *n* = 250; controls *n* = 190; 39.5% female), for whom two consecutive 24-h dietary recalls and biological samples were available. Participants’ characteristics have been previously described [20] and included in Online Resource 1, Supplementary Table 1.

### Dietary data

The dietary data were annotated with levels of live microorganisms and descriptors of fermented foods, including their core microbiota, using the classification approach established for the dataset of the Swiss National Nutrition Survey menuCH 2014–2015, as detailed elsewhere [18].

Briefly, the levels of live microorganisms were chosen to reflect the approximate numbers of viable microorganisms expected to be found in pasteurised foods (Low, < 10^4^ CFU/g), fresh vegetables and fruits consumed unpeeled (medium [Med], 10^4^–10^7^ CFU/g), and unpasteurised fermented foods and probiotics (high [Hi], > 10^7^ CFU/g), taking into account the specific food processing techniques used in Switzerland. For the analysis of live microorganism intake, a combined MedHi category was created by aggregating food items with Med or Hi levels (> 10^4^ CFU/g) [13, 18]. Fermentation status was assigned based on the International Scientific Association for Probiotics and Prebiotics definition of “foods made through desired microbial growth and enzymatic conversions of food components” [25] and included “fermented,” “non-fermented,” or “composite food item with fermented ingredients” categories; while the core microbiota of fermented foods was classified by reviewing 5–10 publications reporting on microbiota for each fermented food type. Foods, beverages and ingredients were aggregated into six main food groups and 35 subgroups (Online Resource 1, Supplementary Table 2). For full details of the dietary data annotation method, refer to the published classification [18].

Additionally, to assess the effects of exposure to the diversity of microorganisms in the diet—in the form of live or inactivated microbes and their metabolites—and to help isolate their effects from the food matrix and other nutrients present, we calculated a microbial species richness index based on the core microbiota identified in fermented foods. For each fermented food item, we used species richness (the count of unique species) as the primary measure of microbial diversity. Where only genus or higher taxonomic group was available, we assigned one species for each of these unique taxonomic groups, assuming that a unique species is present, even though its precise identity is unknown. To account for the impact of consumption quantity on microbial exposure, microbial richness was adjusted based on the total amount of food consumed. For each participant, a weighted microbial richness index was then calculated as follows:

$$ Weighted\,Microbial\,Richness\,Index\,j = \left( {\sum \left( {Qij*Ri} \right)} \right)/\left( {\sum Qij} \right), $$

where *Ri* is the microbial richness of fermented food *i,* and *Qij* is the amount of fermented food *i* consumed by participant *j*.

Furthermore, recognising that health outcomes may also be influenced by the diversity of food subgroups consumed, we computed a dietary diversity index for food subgroups as described in the literature on dietary diversity [26, 27] using Shannon entropy [28]:

$$ Food\,Subgroup\,Shannon\,Index = - \sum \left( {pi*\log \left( {pi} \right)} \right), $$

where *pi* represents the proportion of total gram intake contributed by each food subgroup.

### Physiological and anthropometric health parameters

Parameters linked to cardiometabolic health were selected based on their availability in the SKSC. These included systolic and diastolic blood pressure, blood lipids (high-density lipoprotein [HDL] cholesterol, low-density lipoprotein [LDL] cholesterol, total cholesterol and triglyceride levels), fasting blood glucose and glycated haemoglobin (HbA1c), and anthropometric variables (weight, waist circumference, and body mass index [BMI]). Detailed descriptions of data collection procedures and laboratory methodologies are available in the SKSC study protocol [19].

### Statistical analyses

All statistical analyses were performed using R (version 4.5.0).

Intake of foods containing live microorganisms or fermented foods was assessed in grams and as proportions of total food and beverage intake using mean values from two 24-h dietary recalls. Estimates were summarised with mean, standard deviation (SD), median, and interquartile range (IQR, 25th and 75th percentiles) to describe the variation in the intake distributions. Participants who consumed these foods in at least one recall were classified as consumers. Intake estimates included both consumers and non-consumers, with the proportions of consumers reported. Values for the fermented food microbial richness index and the food subgroup Shannon index were also obtained as the mean of the indices calculated for each of the two 24-h dietary recalls for each participant.

Differences in the estimated intakes of MedHi foods or fermented foods across demographic subgroups were assessed using the Wilcoxon rank sum test to compare intakes by sex, linguistic region, and case–control status, while the Kruskal–Wallis rank sum test was applied for age groups with > 2 subgroups.

Linear regression analyses were used to assess the relationships between the exposures of interest—MedHi foods, fermented foods, and the fermented food microbial richness index—and each of the health parameters, taken one at a time. For each exposure-outcome analysis, we used two adjusted models with a prespecified set of known or potential covariates [14, 29]. Model 1 included age, sex, study centre, case–control status, education level, smoking status, physical activity level, alcohol intake and energy intake. Model 2 included all covariates from Model 1 with the addition of BMI, which is known to affect certain physiological parameters (e.g., glucose and lipids). When the anthropometric measures were the outcomes, only Model 1 was applied. For the fermented food microbial richness index, dietary diversity based on food subgroups (calculated as Shannon entropy) was included as a covariate to control for potential confounding.

Missing values for covariates (education level, *n* = 53; smoking status, *n* = 12) were imputed using the “VIM” R package (version 6.2.2). The K-nearest neighbour algorithm with 10 nearest neighbours was applied using other non-missing covariate data [30, 31]. For outcome variables with missing values, linear regression analyses were performed using available data points, reporting the sample size for each outcome.

Non-normally distributed continuous variables were transformed with the Yeo-Johnson power transformation using the “car” R package (version 3.1–3), with the transformation parameter lambda for each variable reported in Online Resource 1, Supplementary Table 3. The transformation choice was based on the residual diagnostics and its suitability for skewed and zero-inflated distributions [32, 33]. The linear regression models were checked to ensure that their assumptions of linearity, absence of multicollinearity, normality of residuals, and homoscedasticity were satisfied.

We also performed sensitivity analyses to investigate the importance of different food subgroups as mediators of the associations. Sensitivity analyses were completed for associations with a *P* value < 0.05 by adding food subgroups comprising more than 75% of the total gram intake for each predictor variable (for the fermented food microbial richness index, the number of species multiplied by the amount of food consumed) in regression models as covariates. The additional covariates were added sequentially and independently to the models to assess the changes in the associations between exposure variables and health measures. For MedHi foods, these food subgroups were Fruit, Yoghurt and fresh cheese, and Vegetables; for fermented foods—Coffee, Bread products, Beer and cider, and Yoghurt and fresh cheese; and for the fermented food microbial richness index—Coffee, Yoghurt and fresh cheese, Bread products, and Wine.

The level of significance was set at a two-sided *P* value of 0.05.

## Results

Among 440 adults from the SKSC, the mean intake of foods with MedHi live microorganism levels was 6.8% (228.1 g/d) of the total food intake (3476.2 g/d) (Table 1), while the combined mean intake of fermented foods and ingredients accounted for 19.7% (667.3 g/d) of the total food intake (3476.2 g/d) (Table 2). The mean (SD) value for the fermented food microbial richness index was 2.8 (0.8), ranging from 1 to 7.

**Table 1:** Daily intake of foods with live microorganisms in adults in the SKSC

Live microorganism intake^a^	Participants	*N*	Mean (SD)	Median (P25, P75)	Consumers, %^b^	Difference (*P* value)^c^
Total food intake, g/d	All	440	3476.2 (997.9)	3384.4 (2814.8, 3984.6)	100	
Med foods, g/d	All	440	139.5 (124.9)	116.6 (38.1, 207.7)	92.7	
Hi foods, g/d	All	440	88.6 (104.2)	50.0 (10.8, 136.9)	79.3	
MedHi foods, g/d	All	440	228.1 (168.7)	199.6 (99.5, 323.3)	97.7	
Med foods, % daily^d^	All	440	4.1 (3.7)	3.4 (1.2, 5.9)	92.7	
Hi foods, % daily	All	440	2.7 (3.1)	1.7 (0.3, 4.1)	79.3	
MedHi foods, % daily	All	440	6.8 (4.9)	6.0 (2.8, 9.6)	97.7	
MedHi foods, % daily *by sex*	Female	174	7.7 (5.1)	7.1 (4.1, 11.2)	98.3	0.001
	Male	266	6.2 (4.7)	5.5 (2.2, 8.9)	97.4	
MedHi foods, % daily *by age group*	18–34 y.o	73	5.6 (4.4)	4.9 (2.0, 8.2)	94.5	0.006
	35–49 y.o	147	6.1 (4.5)	5.6 (2.3, 8.5)	98.6	
	50–64 y.o	148	7.3 (5.1)	6.3 (3.7, 11.0)	97.3	
	> 65 y.o	72	8.3 (5.4)	7.6 (4.1, 12.7)	100	
MedHi foods, % daily *by linguistic region*^*e*^	German-speaking	242	6.6 (4.9)	5.6 (2.2, 9.3)	97.9	0.24
	French-speaking	198	7.1 (4.9)	6.4 (3.6, 9.9)	97.5	
MedHi foods, % daily *by case–control status*	Case	250	6.7 (5.0)	6.0 (2.5, 9.3)	96.8	0.51
	Control	190	6.9 (4.7)	6.0 (3.2, 9.8)	98.9	

^a^Live microorganism levels: Med, estimated to contain 10^4^–10^7^ CFU/g; Hi, estimated to contain > 10^7^ CFU/g; MedHi, estimated to contain > 10^4^ CFU/g

^b^Participants who reported consuming foods with live microorganisms in at least one of the two 24-h dietary recalls were considered consumers

^c^Differences between the population subgroups were assessed using the Wilcoxon rank sum test for sex, linguistic regions, and case–control status and the Kruskal–Wallis rank sum test for age groups

^d^Proportions of foods with levels of live microorganisms were calculated relative to the total food intake by gram amount for each participant

^e^The German-speaking region included the study centres of Zurich, Basel, Aarau, Bern; the French-speaking region: Lausanne, Geneva

**Table 2:** Daily intake of fermented foods and ingredients in adults in the SKSC

Fermented food intake	Participants	*N*	Mean (SD)	Median (P25, P75)	Consumers, % ^a^	Difference (*P* value)^b^
Total food intake, g/d	All	440	3476.2 (997.9)	3384.4 (2814.8, 3984.6)	100	
Fermented foods and ingredients, g/d	All	440	667.3 (412.1)	601.3 (366.4, 878.1)	100	
Fermented foods and ingredients, % daily ^c^	All	440	19.7 (11.0)	18.1 (11.7, 26.3)	100	
Fermented foods and ingredients, % daily *by sex*	Female	174	17.3 (9.1)	16.0 (10.7, 22.9)	100	0.001
	Male	266	21.3 (11.8)	19.6 (13.0, 28.4)	100	
Fermented foods and ingredients, % daily *by age group*	18–34 y.o	73	13.7 (8.8)	11.7 (7.3, 18.0)	100	< 0.001
	35–49 y.o	147	18.7 (9.5)	17.3 (11.7, 25.0)	100	
	50–64 y.o	148	22.1 (11.9)	19.7 (13.8, 30.2)	100	
	> 65 y.o	72	22.9 (11.2)	22.5 (13.6, 29.8)	100	
Fermented foods and ingredients, % daily *by linguistic region *^*d*^	German-speaking	242	18.9 (10.6)	17.1 (10.8, 26.0)	100	0.10
	French-speaking	198	20.7 (11.3)	19.4 (13.2, 26.7)	100	
Fermented foods and ingredients, % daily *by case–control status*	Case	250	18.5 (9.8)	17.3 (10.8, 24.8)	100	0.03
	Control	190	21.3 (12.2)	19.3 (12.1, 28.5)	100	

^a^Participants who reported consuming fermented foods or ingredients in at least one of the two 24-h dietary recalls were considered consumers

^b^Differences between the population subgroups were assessed using the Wilcoxon rank sum test for sex, linguistic regions, and case–control status and the Kruskal–Wallis rank sum test for age groups

^c^ Proportions of fermented foods and ingredients were calculated relative to the total food intake by gram amount for each participant

^d^The German-speaking region included the study centres of Zurich, Basel, Aarau, Bern; the French-speaking region: Lausanne, Geneva

Several dietary predictors were associated with parameters of cardiometabolic health (Tables 3, 4, 5). Higher MedHi food intake was associated with lower diastolic blood pressure (β = − 1.99 × 10^–2^; CI − 3.71 × 10^–2^, − 2.82 × 10^–3^; *p* = 0.02). While fermented food intake was not associated with any parameter of cardiometabolic health, a higher value for the fermented food microbial richness index was associated with lower systolic blood pressure (β = − 2.07 × 10^–3^; CI − 3.85 × 10^–3^, − 2.95 × 10^–4^; *p* = 0.02) and glycated haemoglobin (β = − 1.22 × 10^–5^; CI − 2.14 × 10^–5^, − 3.11 × 10^–6^; *p* = 0.01). These associations were confirmed in both models, with or without BMI as an additional covariate. There were no other significant associations.

**Table 3:** Adjusted associations of MedHi food intake (per 100 g) with health parameters in adults in the SKSC^a^

Outcome variables	N	Model 1^b^		Model 2^c^	
		Regression coefficient (95% CI)	*P* value	Regression coefficient (95% CI)	*P* value
SBP, mm Hg	426	− 5.64 × 10^–4^ (− 1.70 × 10^–3^, 5.68 × 10^–4^)	0.33	− 4.99 × 10^–4^ (− 1.60 × 10^–3^, 5.98 × 10^–4^)	0.37
DBP, mm Hg	426	− 2.10 × 10^–2^ (− 3.88 × 10^–2^, − 3.29 × 10^–3^)	0.02	− 1.99 × 10^–2^ (− 3.71 × 10^–2^, − 2.82 × 10^–3^)	0.02
Cholesterol, mmol/L	439	1.43 × 10^–2^ (− 2.33 × 10^–2^, 5.19 × 10^–2^)	0.45	1.52 × 10^–2^ (− 2.23 × 10^–2^, 5.28 × 10^–2^)	0.42
HDL, mmol/L	353	7.98 × 10^–3^ (− 5.91 × 10^–3^, 2.19 × 10^–2^)	0.26	6.57 × 10^–3^ (− 6.51 × 10^–3^, 1.96 × 10^–2^)	0.32
LDL, mmol/L	353	− 7.22 × 10^–3^ (− 8.41 × 10^–2^, 6.96 × 10^–2^)	0.85	− 4.58 × 10^–3^ (− 8.10 × 10^–2^, 7.19 × 10^–2^)	0.91
Triglyceride, mmol/L	352	− 1.35 × 10^–2^ (− 2.80 × 10^–2^, 9.37 × 10^–4^)	0.07	− 1.22 × 10^–2^ (− 2.61 × 10^–2^, 1.72 × 10^–3^)	0.09
Glucose (fasting), mmol/L	440	− 3.42 × 10^–5^ (− 4.57 × 10^–4^, 3.88 × 10^–4^)	0.87	− 3.60 × 10^–6^ (− 4.11 × 10^–4^, 4.04 × 10^–4^)	0.99
HbA1c, %	428	4.80 × 10^–7^ (− 5.16 × 10^–6^, 6.12 × 10^–6^)	0.87	5.60 × 10^–7^ (− 4.98 × 10^–6^, 6.09 × 10^–6^)	0.84
Weight, kg	440	5.94 × 10^–3^ (− 1.90 × 10^–2^, 3.09 × 10^–2^)	0.64	NA	NA
BMI, kg/m^2^	440	− 2.37 × 10^–4^ (− 1.13 × 10^–3^, 6.53 × 10^–4^)	0.60	NA	NA
Waist circumference, cm	373	5.75 × 10^–3^ (− 1.41 × 10^–2^, 2.56 × 10^–2^)	0.57	NA	NA

^a^Note that original units for the predictor and outcome variables are reported in the table; however, the non-normally distributed continuous variables were transformed with the Yeo-Johnson power transformation, so the regression coefficient does not represent the unit change. The transformation lambda for each variable is reported in Online Resource 1, Supplementary Table 3

^b^Model 1: age, sex, study centre, case–control status, education level, smoking status, physical activity level, alcohol intake and energy intake

^c^Model 2: model 1 and BMI (except for anthropometric outcome variables)

SBP, systolic blood pressure; DBP, diastolic blood pressure; HDL, high-density lipoprotein cholesterol; LDL, low-density lipoprotein cholesterol; HbA1c, glycated haemoglobin; BMI, body mass index; CI, confidence interval

**Table 4:** Adjusted associations of fermented food and ingredient intake (per 100 g) with health parameters in adults in the SKSC^a^

Outcome variables	N	Model 1 ^b^		Model 2 ^c^	
		Regression coefficient (95% CI)	*P* value	Regression coefficient (95% CI)	*P* value
SBP, mm Hg	426	1.33 × 10^–4^ (− 8.68 × 10^–4^, 1.13 × 10^–3^)	0.79	4.52 × 10^–5^ (− 9.26 × 10^–4^, 1.02 × 10^–3^)	0.93
DBP, mm Hg	426	6.97 × 10^–3^ (− 8.79 × 10^–3^, 2.27 × 10^–2^)	0.39	5.49 × 10^–3^ (− 9.73 × 10^–3^, 2.07 × 10^–2^)	0.48
Cholesterol, mmol/L	439	5.09 × 10^–3^ (− 2.88 × 10^–2^, 3.90 × 10^–2^)	0.77	3.97 × 10^–3^ (− 2.99 × 10^–2^, 3.78 × 10^–2^)	0.82
HDL, mmol/L	353	1.39 × 10^–3^ (− 1.07 × 10^–2^, 1.35 × 10^–2^)	0.82	3.72 × 10^–3^ (− 7.66 × 10^–3^, 1.51 × 10^–2^)	0.52
LDL, mmol/L	353	5.86 × 10^–2^ (− 7.82 × 10^–3^, 1.25 × 10^–1^)	0.08	5.45 × 10^–2^ (− 1.18 × 10^–2^, 1.21 × 10^–1^)	0.11
Triglyceride, mmol/L	352	− 9.28 × 10^–4^ (− 1.34 × 10^–2^, 1.16 × 10^–2^)	0.88	− 2.91 × 10^–3^ (− 1.50 × 10^–2^, 9.15 × 10^–3^)	0.63
Glucose (fasting), mmol/L	440	3.09 × 10^–5^ (− 3.50 × 10^–4^, 4.12 × 10^–4^)	0.87	− 5.88 × 10^–6^ (− 3.73 × 10^–4^, 3.61 × 10^–4^)	0.97
HbA1c, %	428	− 1.19 × 10^–6^ (− 6.30 × 10^–6^, 3.92 × 10^–6^)	0.65	− 1.58 × 10^–6^ (− 6.61 × 10^–6^, 3.44 × 10^–6^)	0.54
Weight, kg	440	1.79 × 10^–2^ (− 4.53 × 10^–3^, 4.04 × 10^–2^)	0.12	NA	NA
BMI, kg/m^2^	440	2.85 × 10^–4^ (− 5.17 × 10^–4^, 1.09 × 10^–3^)	0.49	NA	NA
Waist circumference, cm	373	1.46 × 10^–2^ (− 2.77 × 10^–3^, 3.19 × 10^–2^)	0.10	NA	NA

^a^Note that original units for the predictor and outcome variables are reported in the table; however, the non-normally distributed continuous variables were transformed with the Yeo-Johnson power transformation, so the regression coefficient does not represent the unit change. The transformation lambda for each variable is reported in Online Resource 1, Supplementary Table 3

^b^Model 1: age, sex, study centre, case–control status, education level, smoking status, physical activity level, alcohol intake and energy intake

^c^Model 2: model 1 and BMI (except for anthropometric outcome variables)

SBP, systolic blood pressure; DBP, diastolic blood pressure; HDL, high-density lipoprotein cholesterol; LDL, low-density lipoprotein cholesterol; HbA1c, glycated haemoglobin; BMI, body mass index; CI, confidence interval

**Table 5:** Adjusted associations of fermented food microbial richness index with health parameters in adults in the SKSC^a^

Outcome variables	N	Model 1 ^b^		Model 2 ^c^	
		Regression coefficient (95% CI)	*P* value	Regression coefficient (95% CI)	*P* value
SBP, mm Hg	426	− 2.05 × 10^–3^ (− 3.88 × 10^–3^, − 2.10 × 10^–4^)	0.03	− 2.07 × 10^–3^ (− 3.85 × 10^–3^, − 2.95 × 10^–4^)	0.02
DBP, mm Hg	426	− 2.63 × 10^–2^ (− 5.53 × 10^–2^, 2.63 × 10^–3^)	0.07	− 2.68 × 10^–2^ (− 5.47 × 10^–2^, 1.10 × 10^–3^)	0.06
Cholesterol, mmol/L	439	− 1.86 × 10^–2^ (− 8.12 × 10^–2^, 4.40 × 10^–2^)	0.56	− 1.86 × 10^–2^ (− 8.10 × 10^–2^, 4.38 × 10^–2^)	0.56
HDL, mmol/L	353	1.25 × 10^–2^ (− 9.84 × 10^–3^, 3.48 × 10^–2^)	0.27	1.45 × 10^–2^ (− 6.44 × 10^–3^, 3.54 × 10^–2^)	0.17
LDL, mmol/L	353	8.38 × 10^–2^ (− 3.93 × 10^–2^, 2.07 × 10^–1^)	0.18	8.03 × 10^–2^ (− 4.23 × 10^–2^, 2.03 × 10^–1^)	0.20
Triglyceride, mmol/L	352	− 1.54 × 10^–3^ (− 2.49 × 10^–2^, 2.18 × 10^–2^)	0.90	− 3.38 × 10^–3^ (− 2.59 × 10^–2^, 1.91 × 10^–2^)	0.77
Glucose (fasting), mmol/L	440	− 4.14 × 10^–4^ (− 1.12 × 10^–3^, 2.88 × 10^–4^)	0.25	− 4.14 × 10^–4^ (− 1.09 × 10^–3^, 2.62 × 10^–4^)	0.23
HbA1c, %	428	− 1.20 × 10^–5^ (− 2.14 × 10^–5^, − 2.73 × 10^–6^)	0.01	− 1.22 × 10^–5^ (− 2.14 × 10^–5^, − 3.11 × 10^–6^)	0.01
Weight, kg	440	− 2.48 × 10^–3^ (− 4.40 × 10^–2^, 3.90 × 10^–2^)	0.91	NA	NA
BMI, kg/m^2^	440	4.07 × 10^–6^ (− 1.47 × 10^–3^, 1.48 × 10^–3^)	0.99	NA	NA
Waist circumference, cm	373	− 3.00 × 10^–2^ (− 6.25 × 10^–2^, 2.38 × 10^–3^)	0.07	NA	NA

^a^Note that original units for the predictor and outcome variables are reported in the table; however, the non-normally distributed continuous variables were transformed with the Yeo-Johnson power transformation, so the regression coefficient does not represent the unit change. The transformation lambda for each variable is reported in Online Resource 1, Supplementary Table 3

^b^Model 1: age, sex, study centre, case–control status, education level, smoking status, physical activity level, alcohol intake, energy intake and food subgroup Shannon index

^c^Model 2: model 1 and BMI (except for anthropometric outcome variables)

SBP, systolic blood pressure; DBP, diastolic blood pressure; HDL, high-density lipoprotein cholesterol; LDL, low-density lipoprotein cholesterol; HbA1c, glycated haemoglobin; BMI, body mass index; CI, confidence interval

In the sensitivity analyses (Online Resource 1, Supplementary Tables 4–5), we observed that the association of MedHi foods with diastolic blood pressure was no longer significant after adjusting for fruit and vegetable intake. The associations between the fermented food microbial index and systolic blood pressure and glycated haemoglobin were attenuated by the addition of the tested subgroups, but not dominated by a particular food subgroup.

## Discussion

Our study examined the relationship between the intake of foods with medium or high levels of live microorganisms and fermented foods with a range of physiological and anthropometric parameters of cardiometabolic health among adults in the SKSC. We also introduced a novel metric for capturing the diversity of microbes in fermented foods, the fermented food microbial richness index, which was also investigated in relation to cardiometabolic health measures.

In the SKSC, the consumption of foods rich in microorganisms (MedHi foods) was associated with lower diastolic blood pressure but not with other cardiometabolic parameters. Furthermore, this association appeared to be explained by fruit and vegetable intake. Fermented foods were not directly associated with the cardiometabolic parameters assessed; however, microbial richness in fermented foods was inversely associated with systolic blood pressure and glycated haemoglobin, suggesting that the impact of fermented food on cardiometabolic health may depend on specific microbial characteristics. Interestingly, the robustness of these associations was confirmed in sensitivity analyses for food subgroups.

In a similar analysis by Hill et al., the extensive NHANES dataset was used to associate foods containing live microbes with lower BMI, waist circumference, systolic blood pressure, C-reactive protein, plasma glucose levels, insulin, and triglycerides, as well as higher levels of HDL cholesterol [14]. While we could not confirm the associations of MedHi foods with many of these outcomes, we nevertheless observed a similar association with diastolic blood pressure. The associations in the NHANES dataset between fermented foods and cardiometabolic features were less marked (particularly for plasma glucose and insulin), but they did not consider the importance of microbial diversity in the fermented food effects, which our results indicate may be relevant. The contribution of food subgroups as mediators of the investigated associations was also examined in the NHANES analysis, with some attenuation; whereas in our study, fruit and vegetable intake was found to affect the association between MedHi foods and diastolic blood pressure. The differences observed between the two datasets may be attributed to variations in diet, sample size, or the characteristics of the study populations.

We previously estimated the intake of foods with live microbes and fermented foods in a Swiss nationally representative dataset including adults aged 18 to 75 years (Swiss National Nutrition Survey menuCH 2014–2015), using a similar methodology [18]. As the microbial content of foods is not available in dietary composition databases, this requires additional and detailed classification work. In the SKSC, mean intake of MedHi foods was 6.8% (228.1 g/d) versus 8.0% (269.3 g/d) in menuCH, while fermented food and ingredient intake accounted for 19.7% (667.3 g/d) versus 21.0% (717.1 g/d), respectively. These estimates indicate comparable intakes between the two study populations, with slightly lower relative and absolute intakes in the SKSC. When comparing Swiss and US diets, differences were observed in absolute intake and in food subgroup intake of MedHi foods containing > 10^4^ CFU/g of live microorganisms, with higher intakes in Switzerland [13]. Relative intakes were not reported in the NHANES dataset, but these differences and variations in dietary patterns could, in part, explain the observed differences in the associations with cardiometabolic health.

Existing evidence, including experimental studies in humans, suggests that probiotics offer modest but significant benefits for cardiometabolic outcomes [34–38]. These include improvements in lipid levels, glycemic control, inflammatory markers, and blood pressure, particularly in populations at higher risk for or already suffering from cardiometabolic disorders. Diet has been demonstrated to rapidly and reproducibly reshape the gut microbiome [39], with higher intake of fermented foods associated with increased alpha-diversity and reduced inflammatory markers [5], while lower gut microbiome richness associated with higher adiposity, insulin resistance, dyslipidemia, and inflammation [40]. It has been proposed that the shift to more industrialised and sterile diets could be detrimental to human health [41]. Despite the growing interest in gut microbiota research [42], the classification of whole diets based on their levels of live microbes is relatively new. To our knowledge, this has been done only in the NHANES dataset [13], with several follow-up studies examining various outcomes related to cardiometabolic health, mortality, ageing, and cognitive and mental health [14–17, 43–47].

A challenge in using a combined intake of various MedHi foods and fermented foods is the potential to group foods with opposing effects so that no consistent trends in their associations with health are observed. Different foods have unique matrices and components beyond live or inactivated microbes and their metabolites, including macro- and micronutrients, fibre, and antioxidants, as well as potential contaminants. To better assess the effects of exposure to non-harmful microorganisms, we introduced a fermented food microbial richness index that combines the diversity of species identified in fermented foods with the quantity consumed. We included all live, inactivated, and post-fermentation removed microbes (as a proxy to their metabolites), recognising that each can confer effects on health [25, 48]. Our findings suggest that the diversity of taxa provided by fermented foods is more consistently associated with cardiometabolic outcomes than combining the intake of various microbe-rich foods or fermented foods, and these results were robust to sensitivity analyses. The total intake of MedHi foods or fermented foods in our cohort may be too heterogeneous to capture dietary microbial exposure directly, whereas richness may better reflect the microbial exposure relevant to host-microbe interactions. Mechanistically, a diet with higher microbial richness could enhance short-chain fatty acid production and bile acid signalling, improve endothelial function, and modulate post-prandial glycaemia via effects on incretins and insulin sensitivity [3, 49, 50].

The strengths of this study include the ability to differentiate MedHi food and fermented food intake from microbial richness of fermented foods. This approach allowed to move beyond categorical classifications of “fermented” versus “non-fermented” or broad levels of live microorganisms. The sensitivity analyses we performed improved the interpretability of our findings and support the argument against attributing health effects to a single dominant food subgroup associated with microbial richness in fermented foods. However, the study has limitations. Firstly, the cross-sectional analysis prevents us from making causal inferences. Secondly, the richness index is based on a reference list of core species derived from existing literature and expert knowledge of fermented food production methods, which may not accurately reflect the microbial content of specific products, due to known variations in microbial content between batches of fermented foods [49]. Thirdly, while we controlled for major demographic, dietary, and lifestyle factors, unmeasured variables may still affect the results, leading to residual confounding. Additionally, reverse causation may be a factor: health-conscious individuals might preferentially consume a variety of foods, or intake might be influenced by dietary advice aimed at kidney stone formers. At the time of the study, the SKSC was the only available dataset in Switzerland that combined deep cardiometabolic profiling with dietary data collected via Globodiet®. This dataset allowed us to annotate the descriptors of levels of live microorganisms, fermented foods, and the core microbiota of fermented foods and to examine the effects of these exposures on health-related parameters. However, since our study population consisted of kidney stone formers and healthy controls, the effects we observed may be specific to this study population limiting external validity of the study results, even though we accounted for kidney stone status in our regression analyses. Further research exploring these associations in a larger, representative sample of adults is necessary. Finally, to meet the assumptions of the linear regression models, we applied the Yeo-Johnson transformation to skewed and zero-inflated continuous variables, which limited interpretability of the regression coefficients on the original scale.

Overall, our results provide additional evidence for a connection between dietary microbial diversity and potential cardiometabolic health benefits. In particular, our study suggests that the variety of microorganisms may be more important than merely the quantity of a single food type consumed. Since different microorganisms likely exert varying health effects, it is essential to link the diversity and types of microbes present in food to those found in the gut and their functional outcomes. Future studies should also compare high- versus low-microbial richness within dietary patterns and integrate mechanistic readouts, such as microbiome and metabolomic profiling, to assess the gut microbiota as a functional community and the role of microbial species and their metabolites in health and disease.

In conclusion, microbial diversity in fermented foods appears to be a relevant dimension to consider when exploring the effects of diet-derived microbes on cardiometabolic health.

## Supplementary Information

Below is the link to the electronic supplementary material.

Supplementary Material 1

## Data Availability

Data used for this research are made available upon reasonable request, subject to ethical limitations (swiss-kidney-stone.ch).
