# Supplementary material for: Associations between dietary intake of live microorganisms, fermented foods, and the fermented food microbial richness index and cardiometabolic health in Swiss adults: a cross-sectional analysis
Source: Eur J Nutr. 2026 Feb 12;65(2):51. doi: 10.1007/s00394-026-03895-y (PMC12901115; doi:10.1007/s00394-026-03895-y)
Supplement: Supplementary file 1 — Supplementary Material 1 [file 394_2026_3895_MOESM1_ESM.pdf]

Online Resource 1 to:

Associations between dietary intake of live microorganisms, fermented foods, and the fermented food microbial richness index and cardiometabolic health in Swiss adults: a cross-sectional analysis

European Journal of Nutrition

Eugenia Pertziger [1,2*], Elaine Hillesheim [1], Olivier Bonny [3], Carsten A. Wagner [4], Guy Vergères [1], Murielle Bochud [2] and Kathryn J. Burton-Pimentel [1*]

[1] Agroscope, Food Microbial Systems Research Division, Bern, Switzerland

[2] University of Lausanne, Center for Primary Care and Public Health (Unisanté), Department of Epidemiology and Health Systems, Lausanne, Switzerland

[3] Service of Nephrology, Fribourg State Hospital and University of Fribourg, Fribourg, Switzerland; Service of Nephrology, Lausanne University Hospital, Lausanne, Switzerland

[4] Institute of Physiology and Zurich Kidney Center, University of Zurich, Zurich, Switzerland

* Corresponding authors: Eugenia Pertziger, eugenia.pertziger@unil.ch; Kathryn J. Burton-Pimentel, kathryn.pimentel@agroscope.admin.ch

Supplementary Table 1. Characteristics of participants in SKSC with two 24-h dietary recalls and biological samples available at baseline

**Sample characteristic**	**Case (n = 250)**	**Control (n = 190)**
Age, years	52.1 (13.7)<br>52.0 (42.0, 62.0)	47.0 (13.3)<br>46.0 (36.3, 57.0)
Sex (n)		
Female	87	87
Male	163	103
Linguistic region (n)		
German-speaking	139	103
French-speaking	111	87
Education (n)		
Lower secondary	26	3
Upper secondary	106	48
Tertiary	84	120
Missing	34	19
Smoking (n)		
Non-smoker	151	119
Ex-smoker	20	26
Smoker	70	42
Missing	9	3
Weight, kg	78.6 (16.7)<br>77.9 (68.0, 87.2)	76.1 (15.9)<br>74.0 (63.0, 84.8)
Waist circumference, cm	95.2 (15.0)<br>95.0 (85.0, 104.0)	88.1 (13.8)<br>87.0 (78.0, 96.3)
BMI, kg/m^2	26.5 (5.0)<br>25.6 (23.1, 29.1)	25.2 (4.5)<br>24.4 (22.1, 27.5)
SBP, mm Hg	127.7 (17.5)<br>126.0 (115.1, 137.0)	121.1 (15.7)<br>118.0 (111.5, 130.1)
DBP, mm Hg	80.1 (12.3)<br>80.3 (72.5, 88.4)	75.2 (11.0)<br>75.0 (67.4, 82.5)
Hb1Ac, %	5.4 (0.7)<br>5.3 (5.0, 5.6)	5.3 (0.6)<br>5.3 (5.1, 5.5)
Glucose, mmol/L	5.5 (1.4)<br>5.1 (4.7, 5.7)	5.4 (1.1)<br>5.2 (4.9, 5.6)
Cholesterol, mmol/L	5.0 (1.0)<br>4.9 (4.2, 5.6)	4.9 (1.0)<br>4.8 (4.2, 5.4)
HDL, mmol/L	1.3 (0.3)<br>1.3 (1.1, 1.5)	1.4 (0.4)<br>1.4 (1.2, 1.6)
LDL, mmol/L	3.2 (0.9)<br>3.2 (2.7, 3.8)	3.2 (0.9)<br>3.1 (2.5, 3.8)
Triglyceride, mmol/L	1.4 (0.8)<br>1.1 (0.9, 1.7)	1.2 (0.6)<br>1.0 (0.8, 1.4)
Energy intake, kcal [a]	2108 (682)<br>2054 (1603, 2536)	2140 (655)<br>2106 (1705, 2536)
Alcohol intake, gram [a]	8.1 (16.8)<br>0 (0, 11.0)	13.1 (18.2)<br>7.5 (0, 18.9)

[a] Calculated using the mean intake of the two 24-h dietary recalls for each participant.

Values are Mean (SD), Median (25th, 75th), or number of participants (n).

BMI, body mass index; SBP, systolic blood pressure; DBP, diastolic blood pressure; Hb1Ac, glycated haemoglobin; HDL, high-density lipoprotein cholesterol; LDL, low-density lipoprotein cholesterol.

Supplementary Table 2. Food groups and subgroups

Food Groups	Food Subgroups
1. Non-caloric beverages	1.1 Water
	1.2 Tea
	1.3 Coffee
2. Fruit & vegetables	2.4 Vegetables
	2.5 Fruit
	2.6 100% fruit and vegetable juices
3. Cereal products & potatoes	3.7 Tuber products
	3.8 Bread products
	3.9 Pasta & rice
	3.10 Other cereal products
4. Protein-based products	4.11 Milk
	4.12 Yoghurt & fresh cheese
	4.13 Soft cheese
	4.14 Hard cheese
	4.15 Red meat
	4.16 Other unprocessed meat
	4.17 Processed meat
	4.18 Fish & seafood
	4.19 Other protein-based products
5. Added fats & nuts	5.20 Vegetable oil
	5.21 Butter
	5.22 Cream, fatty sauces & other fats
	5.23 Nuts, seeds & olives
6. Sweets, salty snacks & alcohol	6.24 Added sweeteners
	6.25 Cakes, desserts & ice cream
	6.26 Chocolate products
	6.27 Other sweet products
	6.28 Salty snacks
	6.29 Soft drinks
	6.30 Beer & cider
	6.31 Wine
	6.32 Other alcohols
	6.33 Fortified wines, liqueurs & spirits
	6.34 Condiments & seasonings
	6.35 Artificial sweeteners

Supplementary Table 3. Yeo-Johnson transformation parameter lambda for continuous variables in linear regression analyses

Variable	Lambda
Age, years	0.570
Energy intake, kcal/day	0.617
Alcohol intake, g/day	-0.292
BMI, kg/m2	-1.017
Weight, kg	-0.032
Waist circumference, cm	-0.036
SBP, mm Hg	-0.583
DBP, mm Hg	-0.056
Hb1Ac, %	-4.093
Glucose (fasting), mmol/L	-2.098
Cholesterol, mmol/L	0.211
HDL, mmol/L	-0.685
LDL, mmol/L	0.459
Triglyceride, mmol/L	-1.411
MedHi food intake, per 100 g	0.161
Fermented food and ingredient intake, per 100 g	0.222
Fermented food microbial richness index	0.456
Food subgroup Shannon index	1.968

BMI, body mass index; SBP, systolic blood pressure; DBP, diastolic blood pressure; Hb1Ac, glycated haemoglobin; HDL, high-density lipoprotein cholesterol; LDL, low-density lipoprotein cholesterol; MedHi, estimated to contain >10^4 CFU/g.

Supplementary Table 4. Adjusted sensitivity analyses for the MedHi food intake predictor [a]

Outcome variables	Additional covariate [b]	N	Model 1 [c]		Model 2 [d]	
			Regression coefficient (95% CI)	P value	Regression coefficient (95% CI)	P value
DBP	Fruit	426	-7.75×10^{-3} (-2.99×10^{-2}, 1.44×10^{-2})	0.49	-6.58×10^{-3} (-2.80×10^{-2}, 1.48×10^{-2})	0.55
DBP	Yoghurt & fresh cheese	426	-3.66×10^{-2} (-5.83×10^{-2}, -1.50×10^{-2})	0.001	-3.46×10^{-2} (-5.55×10^{-2}, -1.37×10^{-2})	0.001
DBP	Vegetables	426	-1.44×10^{-2} (-3.40×10^{-2}, 5.24×10^{-3})	0.15	-1.29×10^{-2} (-3.19×10^{-2}, 5.99×10^{-3})	0.18

[a] Note that original units for the predictor and outcome variables are reported in the table; however, the non-normally distributed continuous variables were transformed with the Yeo-Johnson power transformation, so the regression coefficient does not represent the unit change. The transformation lambda for each variable is reported in Supplementary Table 3.

[b] Additional covariates were added one at a time to assess the change in the association of exposure variables with health parameters.

[c] Model 1: age, sex, study centre, case-control status, education level, smoking status, physical activity level, alcohol intake, energy intake and additional covariate.

[d] Model 2: model 1 and BMI (except for anthropometric outcome variables).

DBP, diastolic blood pressure; CI, confidence interval; BMI, body mass index.

Supplementary Table 5. Adjusted sensitivity analyses for the fermented food microbial richness index predictor [a]

Outcome variables	Additional covariate [b]	N	Model 1 [c]		Model 2 [d]	
			Regression coefficient (95% CI)	P value	Regression coefficient (95% CI)	P value
SBP	Coffee	426	-2.18×10^{-3} (-4.37×10^{-3}, 1.80×10^{-5})	0.05	-2.14×10^{-3} (-4.26×10^{-3}, -9.00×10^{-6})	0.05
SBP	Yoghurt & fresh cheese	426	-2.09×10^{-3} (-3.93×10^{-3}, -2.39×10^{-4})	0.03	-2.11×10^{-3} (-3.89×10^{-3}, -3.15×10^{-4})	0.02
SBP	Bread products	426	-1.61×10^{-3} (-3.46×10^{-3}, 2.43×10^{-4})	0.09	-1.69×10^{-3} (-3.48×10^{-3}, 1.13×10^{-4})	0.07
SBP	Wine	426	-2.05×10^{-3} (-3.89×10^{-3}, -2.11×10^{-4})	0.03	-2.07×10^{-3} (-3.85×10^{-3}, -2.89×10^{-4})	0.02
Hb1Ac	Coffee	428	-1.32×10^{-5} (-2.42×10^{-5}, -2.19×10^{-6})	0.02	-1.30×10^{-5} (-2.38×10^{-5}, -2.19×10^{-6})	0.02
Hb1Ac	Yoghurt & fresh cheese	428	-1.06×10^{-5} (-2.00×10^{-5}, -1.23×10^{-6})	0.03	-1.08×10^{-5} (-2.00×10^{-5}, -1.61×10^{-6})	0.02
Hb1Ac	Bread products	428	-1.11×10^{-5} (-2.06×10^{-5}, -1.69×10^{-6})	0.02	-1.15×10^{-5} (-2.08×10^{-5}, -2.19×10^{-6})	0.02
Hb1Ac	Wine	428	-1.17×10^{-5} (-2.11×10^{-5}, -2.39×10^{-6})	0.01	-1.19×10^{-5} (-2.11×10^{-5}, -2.75×10^{-6})	0.01

[a] Note that original units for the predictor and outcome variables are reported in the table; however, the non-normally distributed continuous variables were transformed with the Yeo-Johnson power transformation, so the regression coefficient does not represent the unit change. The transformation lambda for each variable is reported in Supplementary Table 3.

[b] Additional covariates were added one at a time to assess the change in the association of exposure variables with health parameters.

[c] Model 1: age, sex, study centre, case-control status, education level, smoking status, physical activity level, alcohol intake, energy intake and additional covariate.

[d] Model 2: model 1 and BMI (except for anthropometric outcome variables).

SBP, systolic blood pressure; Hb1Ac, glycated haemoglobin; CI, confidence interval; BMI, body mass index.
